# Fertility preservation in Malaysian pediatric cohort: a survey of healthcare providers’ knowledge, practice, attitude, perceptions and barriers

**DOI:** 10.3389/fped.2024.1419515

**Published:** 2024-09-19

**Authors:** Anizah Ali, Yew Kong Lee, Hamidah Alias, Ani Amelia Zainuddin

**Affiliations:** ^1^ Department of Obstetrics and Gynaecology, Faculty of Medicine, Universiti Kebangsaan Malaysia (UKM), Kuala Lumpur, Malaysia; ^2^ Paediatrics and Adolescent Gynaecology (PAG) Unit, Department of Obstetrics and Gynaecology, Faculty of Medicine, Universiti Kebangsaan Malaysia (UKM), Kuala Lumpur, Malaysia; ^3^ Department of Primary Care Medicine, Faculty of Medicine, University of Malaya, Kuala Lumpur, Malaysia; ^4^ Department of Paediatric, Faculty of Medicine, Hospital Tunku Ampuan Besar Tuanku Aishah Rohani, Universiti Kebangsaan Malaysia (UKM), Kuala Lumpur, Malaysia

**Keywords:** fertility preservation, knowledge and practice, attitude and perception, barriers, children and adolescents, healthcare providers (HCPs), survey, Malaysia

## Abstract

**Introduction:**

Impaired future fertility potential secondary to gonadotoxic therapies for childhood cancer is a shattering aftermath faced by childhood cancer survivors. Fertility preservation (FP) has emerged as a key to mitigate this unwelcomed sequelae. FP services catering to the needs of children and adolescents (C&A) population in developing countries are limited. Malaysia recently launched its pioneering pediatrics FP services.

**Aims of study:**

To evaluate healthcare providers’ (HCPs) FP knowledge, practice behaviors, attitudes, perceptions, and barriers towards FP counseling/services (C/S) for the C&A cohort.

**Methods:**

A questionnaire-based study was conducted utilizing a questionnaire consisting of 51 items which was adapted from G.Quinn et al. The questionnaire was distributed both online and physically amongst HCPs in a tertiary center. Ethical committee approval was granted by the Research Ethical Committee, Universiti Kebangsaan Malaysia.

**Results:**

A total of 102 HCPs completed the questionnaires. The majority of respondents were Malays (74.5%), females (80.4%), gynecology/pediatrics specialty (76.5%), and had children (88.2%). Nearly 72% of HCPs demonstrated good knowledge of FP. Almost 73% of HCPs consulted reproductive specialists (RES) on potential fertility issues and over 80% of HCPs referred patients who enquired on fertility issues to RES. Only 17% of HCPs practiced FP discussion, 12% reported no available person to discuss FP, and 10% of HCPs were unaware of who to discuss FP with. Patients’ inability to afford FP (30.4%) tops the list of barriers to FP C/S, followed by limited available information on FP for patients (17.6%) and patients too ill to delay treatment (12.7%). Most HCPs (88.2%) demonstrated unfavorable attitudes towards FP C/S.

**Discussions:**

In general, the majority of our HCP respondents demonstrated good current FP knowledge and practice behaviors. Mitigating several controversial issues in FP would improve HCPs’ attitude towards FP. Main barriers to the uptake of FP C/S for C&A were patient and resource barriers. Addressing these issues by funding aid for FP procedures, increasing FP knowledge dispersion, as well as developing age-appropriate FP-related educational materials would improve FP service provision for C&A in the future.

**Conclusions:**

In conclusion, successful corrective action combined with strategic planning points to a promising future for Malaysia's FP services provision for C&A.

## Introduction

Advancements in medical diagnosis and treatment modalities such as the combination of chemotherapy, radiotherapy, and multimodality cancer treatments have notably increased the overall survival rates for pediatric cancer patients, reaching nearly 80%. More childhood cancer survivors are now maturing and entering life as adolescents and soon-to-be adults. Nevertheless, while rejoicing in survivorship, these childhood cancer survivors may be faced with long-term cancer treatment sequelae, especially fertility impairment (1, 2). This fertility impairment has a profound effect on their emotional well-being as well as psycho-social functioning (3–5).

Fertility preservation has emerged as a potential solution to combat fertility impairment risk among childhood cancer patients undergoing cancer treatment. Fertility preservation is a term describing the process of safeguarding a child's or adolescent's ability to reproduce before undergoing any medical treatments or procedures that could potentially impact or reduce their future fertility potential (6, 7). Fertility preservation techniques for adults are more established and widely accessible. Considering fertility preservation techniques for female children and adolescents, the options are limited to oocyte cryopreservation [a process where a female's mature eggs (oocytes) are extracted, frozen, and stored, to be used in the future for conception via assisted reproductive techniques]. Another C&A FP option is ovarian tissue cryopreservation [a process whereby the female ovary is surgically removed and subsequently processed into small strips which are then frozen to preserve them. This stored ovarian tissue allows for potential future re-implantation to restore fertility needs]. The feasibility of any FP method for C&A patients would be dependent on their pubertal status and time before cancer treatment commencement (8). Gonadotoxic [irreversible damage to the ovaries in females and testes in males, resulting in impaired future fertility potential] cancer therapy effects are well documented in the literature. Acknowledging the critical need to address FP in patients undergoing such treatments, the American Society of Clinical Oncology (ASCO) issued guidelines in 2006. These guidelines recommend that healthcare providers initiate discussion on the potential cancer treatment risks to fertility with patients, and refer them accordingly to reproductive specialists for FP counseling before the start of cancer treatment (9).

Although fertility preservation has grown much throughout the world, substantial progress remains necessary, particularly in ASEAN countries, to enhance the availability as well as quality of FP services for C&A. While there has been a growing understanding of the significance of fertility preservation for this group, many healthcare facilities are not yet equipped to provide specialized and comprehensive fertility care for pediatric patients. Of date, the availability of fertility preservation services catering to children and adolescents in the ASEAN region is still limited. Among countries that have started such services, it may be confined to only a few centers (10).

Similarly, fertility preservation services for children and adolescents are still relatively new in Malaysia. Its first oncofertility center was launched in late 2020, which caters to oncofertility needs for adults and children alike, and all sexes. Embarking on a journey offering such new services, commitment, due acceptance, and uptake by the healthcare providers managing eligible patients is the key to successful service provision. Healthcare providers’ pivotal role in support of this new oncofertility services cannot be overstated. Healthcare providers’ approaches and views regarding these oncofertility services can greatly influence the conduct of FP services, discussions, and counseling offered to the C&A cohort. Thus, it is essential to gain a deeper insight into healthcare providers’ acceptance of FP counseling/services for C&A. This knowledge is crucial in enhancing the provision of comprehensive care for this vulnerable population (11). Therefore, a survey was conducted to understand and assess healthcare providers’ (HCPs) knowledge, practice behaviors, barriers, attitudes, and perceptions toward FP counseling/services for C&A.

## Methods

This study utilized a cross-sectional design whereby a questionnaire survey was conducted to evaluate the knowledge, practices, attitudes, barriers, and perceptions of fertility preservation services and counseling for C&A amongst HCPs in a tertiary medical center. The questionnaire was disseminated both electronically through email correspondence conducted online and in hard copy form.

### Survey instrument adaptation

A pre-validated questionnaire, formerly used to assess oncologists’ beliefs and practices related to FP in cancer patients in the United States (12) was modified and adapted; with the authors’ consent. The questionnaire was adapted to explore and measure self-reported knowledge, practices, attitudes, barriers, and perceptions concerning FP services and counseling for children and adolescent cohorts among healthcare practitioners. If deemed appropriate, similar items of the original questionnaire were maintained, while some adaptations were made to suit our local Malaysian context.

The instrument adaptation incorporated content validity assessment, which involved expert panels giving feedback and comments throughout the process. These expert panels comprised pediatric oncologists, fertility and reproductive specialists, primary care experts, and researchers with experience in questionnaire design and fertility preservation. Findings from previous literature on FP for C&A were also sought to guide the adaptation process. The expert panel reviewed the original questionnaire and then adapted items according to established guidelines for items review; whereby emphasis was on aspects of (i) information clarity; ensuring that the items were worded clearly without any confusing terminologies or ambiguity in terms of phrases or language used. The next aspect scrutinized was (ii) the relevance of items to Malaysian local practices and cultural context- which also encompassed adaptations of answer options to be all-inclusive to our local practices. Care was also taken to avoid highly sensitive issues which may not be well accepted locally. Lastly, was the aspect of (iii) coverage of all pertinent aspects with regards to FP for C&A in the context of Malaysian HCP practices in a center that commence its oncofertility services fairly recent. We abided by the best practices in this questionnaire adaptation process. Following this, the adapted questionnaire underwent pilot testing which will be elaborated below.

The final adapted version of the questionnaire (see Supplementary Material) retained the key components and themes identified through extensive preliminary research, including these five domains: (1) FP knowledge; (2) FP practice behaviors; (3) barriers to FP; (4) attitudes and perceptions towards FP; and (5) demographic and practice information. The final section of the questionnaire was dedicated to demographics and medical practice background data of respondents (ethnicity, religious background, gender, year graduate, years in practice, specialty, primary practice location/situation and settings, number of oncology patients/patients age 0–30 per week, membership of the professional organization, having any children, close family members experiencing cancer and motivation to be a healthcare provider).

The majority of responses were provided on a Likert scale, which measured the level of agreement with a set of given statements (strongly agree to strongly disagree/always to never/don't know). Participants were prompted to answer based on their firsthand experience concerning FP services and counseling provision among their patients or for those not dealing with eligible patients, their responses would be based on their knowledge or opinions of FP service provision in their practice centers. A free-text box was included at the end of the questionnaire to enable respondents to add in or give any comments with regards to FP for C&A.

### Measures

The adapted version of the questionnaire generally maintained the domains measured in the original version, with minor adjustments in terms of answer options as HCPs in our pioneering oncofertility center were still relatively new to this FP services compared to oncologists’ cohort in the original study.

#### Knowledge on FP

Under the domain of (A) Knowledge on FP there were 5 knowledge-based statements meant to evaluate respondents’ agreement with the statements. Respondents were required to specify their agreement to the given statements via Likert scale items (strongly agree to strongly disagree). An example of the question in this domain is as follows:

A1*:* “Alkylating agents have been linked to infertility in cancer patients”

All responses were scored and the mean total score for each knowledge item was obtained. A cut-off means a total score of ≥4 was used to categorize respondents as “Knowledgeable”, while those with a mean total score of <4 were categorized as “Not Knowledgeable”. This mean total score cut-off points were similar to that used by the author of the original questionnaire.

#### FP practice behaviors

This second domain (B) FP Practice Behavior had a total of 3 statements which all respondents were required to attend to. For the two initial statements, respondents signified their agreement with each statement using Likert-scale items (always to never). The mean total score of respondents’ responses was calculated and again cut-off mean total score of ≥4 was used to categorize HCPs into those with “Good Practice Behavior” and those with a mean total score of <4 as “Poor Practice Behavior”. An example of the statement given was:

B2: “I refer patients who have questions about fertility to an infertility specialist or reproductive endocrinologist”

The third statement which all respondents need to respond to assessed who in their respective practices conducted FP discussions with patients and how are their (HCPs) FP referral practices (where applicable). Respondents needed to choose from a list of responses provided. Responses that were in support of FP practices/referrals were categorized as “Good Practice Behavior”.

To aid analysis and for clearer representation of the data, Responses to statement B2a were re-grouped into 4 new groups; (i) Myself and other colleague, (ii) Someone else, (iii) Unaware of who to discuss/refer for FP, and (iv) None available. The former two groups indicated “Good Practice Behavior” while the balance was “Poor Practice Behavior”. Total scores of statements B1, B2, and B2a were calculated, and an average value was obtained. The mean total score of ≥4 was used to categorize overall HCPs’ practice behaviors into those with “Good Practice Behavior”.

#### Barriers to FP

In this domain, all respondents were asked to identify barriers to FP practices. HCPs who did not practice FPC were asked to identify perceived primary barriers to FP practices from a list provided (C6a). The initial barrier list from the original questionnaire was adapted by adding three additional barrier options to the listing which were “I am not comfortable to discuss fertility preservation with my patients.”, “healthcare providers having limited knowledge to indulge in FP discussion” and “healthcare providers unaware of the availability of FP services”. The first addition was included as physicians’ discomfort was identified among the main barriers to FP discussion in prior publications (13). On the other hand, the latter two additional options were important to assess as they would reflect current gaps in FP information and awareness of FP services availability which are both valuable information for the improvement of FP services/counseling practices. Respondents were expected to indicate their agreement with the given statements via Likert-scale items (Always to Never).

#### FP attitudes

Only five out of the original six statements were incorporated to assess respondents’ attitudes and perceptions toward FP practice and discussion. An item was omitted as it involved diseases affecting adult women mainly and involving procedures which was not widely offered. [e.g., “Some patients with certain cancers (e.g., hereditary breast and ovarian cancer) should be informed about preimplantation genetic diagnosis (PGD)”]. Respondents were required to indicate their agreement to the given statements using Likert-scale items (Strongly agree to strongly disagree). All respondents were assessed with regard to their attitude towards FP practices. Initially, 5 statements were included in this FP Attitude domain, however, a single statement (D3) regarding posthumous parenting was omitted from the analysis due to the perceived sensitivity of the statement in the local setting. Thus, the final four statements assessed in this domain were D1, D2, D4, and D5. Respondents were regarded to have an overall favorable attitude toward FP practices using an average attitude score of 4 as proposed by the original questionnaire (12). An example of a statement assessed in this domain is as follows:

D1: “Patients with a poor prognosis should not pursue fertility preservation”

#### FP perceptions

Respondents were asked to specify based on their experience in practice, perceptions of patients’ interest in FP based on the patient's degree of concern and according to patient's ethnicity, socioeconomic status, and gender (e.g., “Female patients are more concerned about FP than male patients”). Respondents who were not practicing or exposed to FP practices were asked to indicate their opinions on similar statements. We used all four statements from the original questionnaire without any adaptations. Respondents indicated their agreement to the statements via Likert-scale items (always to don't know) in three items. The Likert-scale options for the first statement which originally was (always to never) were adapted with the addition of another option of “I do not manage infertile OR cancer patients in my practice”.

#### Socio-demographic profile, medical training backgrounds, and clinical practice characteristics

Demographic information that was enquired of the respondents included Socio-demographic Profile and Medical Training Background; which included ethnicity, religious background, gender, parental status, close family members who experienced cancer, motivation to become healthcare provider, year of graduation from medical school, specialty, and membership of professional organizations. Clinical Practice Characteristics were also assessed which encompassed the number of years in practice, practice location, practice situation, size of practice setting, and volume of patients who were seen typically per week, patients’ ages, and gender.

### Procedures

To ensure clarity and sound interpretation of the adapted questionnaire, the survey instrument was piloted among 10 HCPs in the center who were not involved in the study. This was to assess the ease of comprehension, the content, and acceptability of our adapted questionnaire. Additionally, this also enabled us to gauge the time needed to complete the questionnaire. The expert panels again reviewed the pilot test findings, taking note of areas which need improvements and amendments to improve comprehension and acceptability of the adapted questionnaire. In terms of missing FP content aspect, the existing questionnaire had covered most of the pertinent issues on C&A oncofertility, thus there was not much concern arising with regards to missing key topics. Overall respondents were able to grasp the questions posed as per the intended survey design. We did not incorporate any cognitive interviewing during evaluation of the pilot test results, as the written feedbacks received from a few respondents in the open box provided were clearly elaborative of the issues which needs to be addressed in the items. The researcher did contact these respondents to verify their feedbacks and gained clarity on the matter prior to amendment. The items were subsequently amended as per suggestions to enable those who did not directly practice FP counseling/services to be able to meaningfully contribute to the study. These are namely re-phrasing of a few items as they appeared to be non-representative of the diverse specialty of HCPs. These involved statements in the Clinical Practice Characteristics, Practice Behavior, and Barrier domains. Overall, the pilot survey was able to be completed in under 40 min. A text box was also provided at the end of the questionnaire to collect open comments and inputs on FP from respondents. The final adapted version of the survey instrument comprised 51 items and took 15–25 min to complete. Reliability testing was also performed. The Cronbach alpha score obtained by the adapted questionnaire was 0.717.

 The institutional review board permitted us to perform this study, and the methods we used complied with the committee's ethical guidelines. A list of potential respondents was created by a compilation of HCPs from several departments in a tertiary teaching hospital in Kuala Lumpur. Three departments involved were Obstetrics and Gynecology, Pediatrics, and Oncology as the doctors from these departments were directly involved in caring for children and adolescent patients. The pediatric surgical unit which was under the Surgical department was also included. All levels of healthcare providers were included i.e., Medical Officers, Fellows, Specialists, and Consultants. Names, email addresses, and contact numbers of these potential respondents were obtained from the respective departments’ offices. Once the list was compiled, the names were randomized using computer-generated randomization.

Emails containing links to the questionnaire which includes the description of the study, consent form, and finally the questionnaire were sent to all 240 respondents. A first reminder email was sent approximately 2 weeks following the initial invite email. A second reminder email was sent a month after the initial email. Printed-out versions of the questionnaires were also sent out to the respondents following the second reminder email.

### Data analysis methods

Analysis was conducted using a standard statistical software package (SPSS, version 29.0). The primary analysis was descriptive. Healthcare providers’ demographic details along with specialty characteristics were summarized in frequencies and proportions.

## Results

### Response rate

In total, questionnaires were distributed via e-mails and printed out hard copies to 240 potential HCP respondents. A total of 102 respondents completed the questionnaire (*N* = 102). There were however 2 undelivered e-mails and a single printed-out questionnaire which was incomplete. The response rate was calculated by dividing the number of respondents who completed the questionnaire (*n* = 102) by the initial number of respondents in the sample minus responses that were considered ineligible and undelivered questionnaires [240—(1 + 2)]. This generated a response rate of 43%.

### Sociodemographic profile and medical training background

Respondents were largely of Malay ethnicity, females, Muslims, had children, and had positive self or close family members who were diagnosed with cancer. Since the median for the Year of graduation from medical school was 2009.87 + 0.576, it was regrouped into (i) the year 2010 or earlier and (ii) the year 2011 or later to aid analysis. Nearly 60% of HCPs who responded graduated from medical school in year 2011 or later.

### Clinical practice characteristics

Most HCPs primarily served in university-affiliated or teaching hospitals. The majority of our healthcare providers were specialized in gynecology and served in a multi-specialty type of practice and most worked in a large practice setting with more than 16 physicians practicing in the center. Healthcare providers who see oncology patients on a weekly basis exceeded 95%. Majority of the HCPs; 70 (68.6%) saw only female patients while 17 HCPs (16.7%) saw majority of female patients. Tables 1, 2 detailed the respondents’ sociodemographic profile, medical training background, and clinical practice characteristics.

**Table 1:** Sociodemographic profile and medical training background of overall HCPs.

Sociodemographic profile and medical training		
Background	*N*	%
Ethnicity		
Malay	76	74.5%
Chinese	14	13.7%
Indian	12	11.8%
Religion		
Islam	77	75.5%
Buddha	11	10.8%
Christian	6	5.9%
Hindu	8	7.8%
Gender		
Male	20	19.6%
Female	82	80.4%
Year graduated from medical school		
2010 or earlier	41	40.2%
2011 or later	61	59.8%
Specialty		
Pediatric	6	5.9%
Gynecology	72	70.6%
Oncology	15	14.7%
Others	9	8.8%
Membership of professional organization		
Yes	90	88.2%
No	12	11.8%
Have children		
Yes	90	88.2%
No	12	11.8%
Have either self/close family members experienced cancer		
Yes	56	54.9%
No	46	45.1%
Motivation to become healthcare provider		
Personal or family history of cancer/interest in cancer or cancer research	47	46.1%
Non-cancer related	55	53.9%

**Table 2:** Clinical practice characteristics of overall HCPs.

Clinical practice characteristics	*N*	%
Years in practice		
≤15 years	80	78.4%
>15 years	22	21.6%
Primary practice location		
Government Hospital/clinic	17	16.7%
Private practice	4	3.9%
University-affiliated/teaching hospital	81	79.4%
Practice type		
Single speciality	17	16.7%
Multi-speciality	85	83.3%
Size of practice settings		
Medium (2–15 physicians)	39	38.2%
Large (≥16 physicians)	63	61.8%
Number of oncology patients seen per week		
10 or less	81	79.4%
11–25	14	13.7%
26–50	1	1.0%
More than 50	1	1.0%
Do not see any oncology patients	5	4.9%
Number of patients aged 0–30 seen per week		
10 or less	68	66.7%
11–25	19	18.6%
26–50	4	3.9%
More than 50	7	6.9%
Do not see any patients aged 0–30	4	3.9%
Gender of patients aged 0–30 seen per week		
Females only	70	68.6%
Majority females	17	16.7%
Majority males	4	3.9%
Equal number of male and female	7	6.9%
None seen in this age range	4	3.9%

### Fertility preservation knowledge

**Figure 1:**
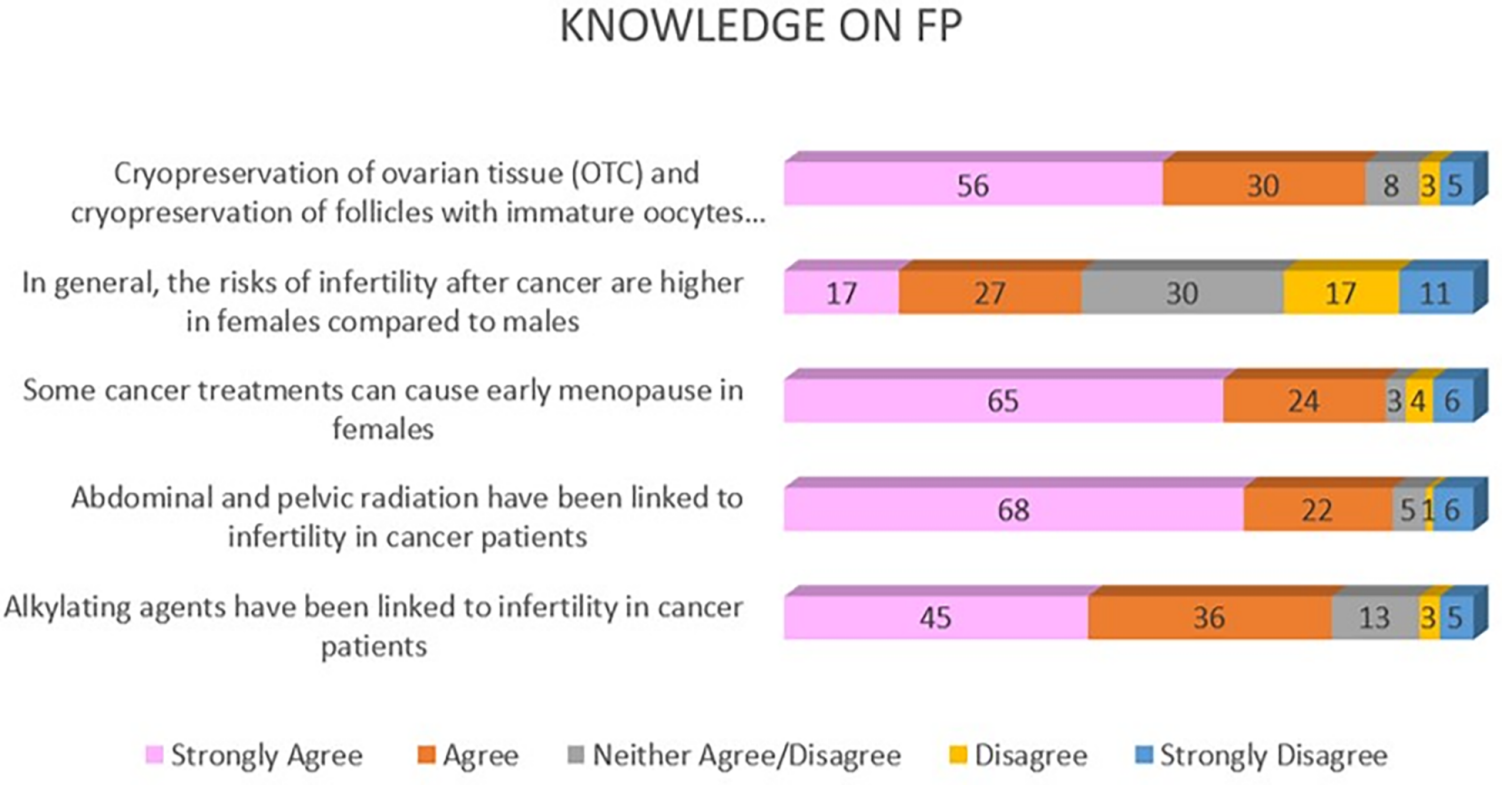
Knowledge of female FP among HCPs.

Under the domain of FP knowledge, there were 5 statements (A1–A5). Overall the HCPs demonstrated good knowledge of female FP. The majority of HCPs identified that alkylating agents, abdominal and pelvic radiation have been linked to infertility in cancer patients following treatment. A single statement, however, revealed that the vast majority of the respondents were not knowledgeable in terms of infertility risks following cancer treatment among different sexes. Most HCPs either were either in disagreement with the statement that risk of infertility after cancer is higher in females compared to males, or choose to neither agree/disagree, which contributed to higher rates of not knowledgeable responses for this item (see Figure 1). Regardless, 73 HCPs (71.6%) demonstrated overall good knowledge of FP (see Table 3).

**Table 3:** FP knowledge, attitude and perception domain status of overall HCPs.

Variables	*N*	%
FP Knowledge		
Not knowledgeable	29	28.4
Knowledgeable	73	71.6
Practice behavior		
Poor practice behavior	37	36.3
Good practice behavior	65	63.7
Attitude		
Unfavorable attitude	90	88.2
Favorable attitude	12	11.8

### Fertility preservation (FP) practice behavior of overall respondents

Fertility Preservation (FP) practice behavior amongst HCPs was assessed via three general statements (B1, B2, and B2a).

B1: “I consult an infertility specialist or reproductive endocrinologist with questions about potential fertility issues”

B2: “I refer patients who have questions about fertility to an infertility specialist or reproductive endocrinologist.”

B2a: “Someone else within my practice discusses fertility preservation (FP) with patients”

Generally, the vast majority of HCPs showed due consideration to FP in their practice as demonstrated by “Good Practice Behavior” for items B1 & B2 exceeding 70% and surpassing 80%; respectively. A few HCPs shared that they “never” needed to consult an infertility specialist or reproductive endocrinologist of any potential infertility issues as their practices neither involve patients who are newly diagnosed with cancer nor those with infertility issues; (12.7% for item B1 and 9.8% for item B2). On the other hand, 15 HCPs (14.7%) volunteered that they “rarely” consult an infertility specialist or reproductive endocrinologist despite having had questions about potential infertility issues. A similar trend was seen for item B2 but at a slightly lower figure of 7 HCPs (6.9%).

**Figure 2:**
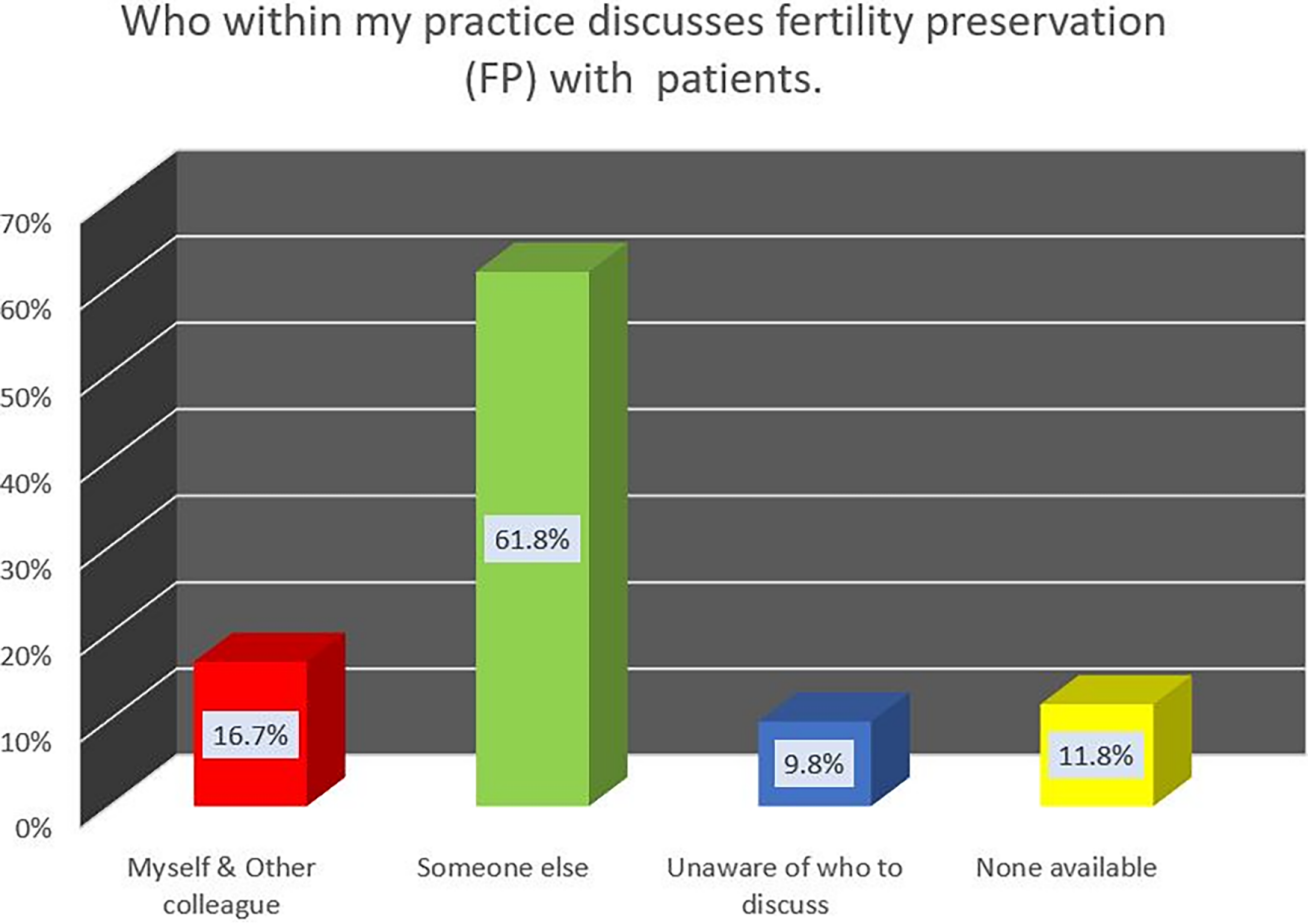
Practice of discusssing FP with patients.

In response to item B2a, largely, 85 HCPs (83.4%) were not personally involved in conducting FP discussions with patients. Sixty-three HCPs indicated that someone else in their practice conducts the FP discussions with patients; whereby they refer suitable patients to these personnel for FP discussions. FP discussion practice are depicted in Figure 2.

### Barriers to fertility preservation (FP) practices/discussions

HCPs were required to choose a single primary barrier towards FP practices/discussions. The top three barriers towards FP practices/discussions as voted by HCPs were “patients” inability to afford FP’ (30.4%). This was followed by “limited available information on FP to give patients” (17.6%), and “patient too ill to delay treatment” (12.7%). An interesting response that was received under the “Others” barrier was the remarks of:

“I treat gynaecological malignancy”.

**Figure 3:**
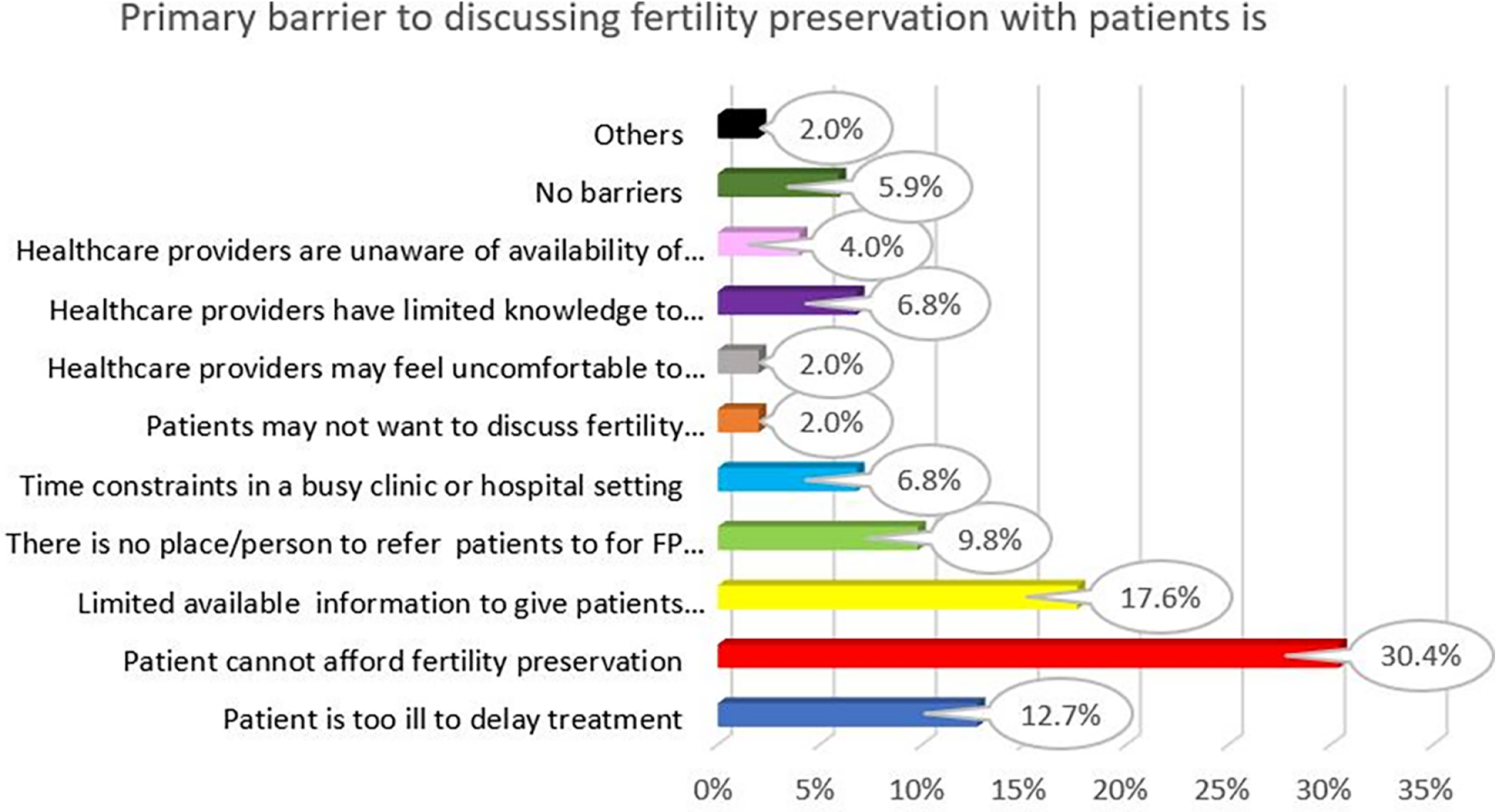
Primary barriers to FP practice/discussion.

Figure 3 depicts the primary barriers to FP practices/discussions as voted by all HCP respondents.

### Attitude towards fertility preservation (FP) practice

**Figure 4:**
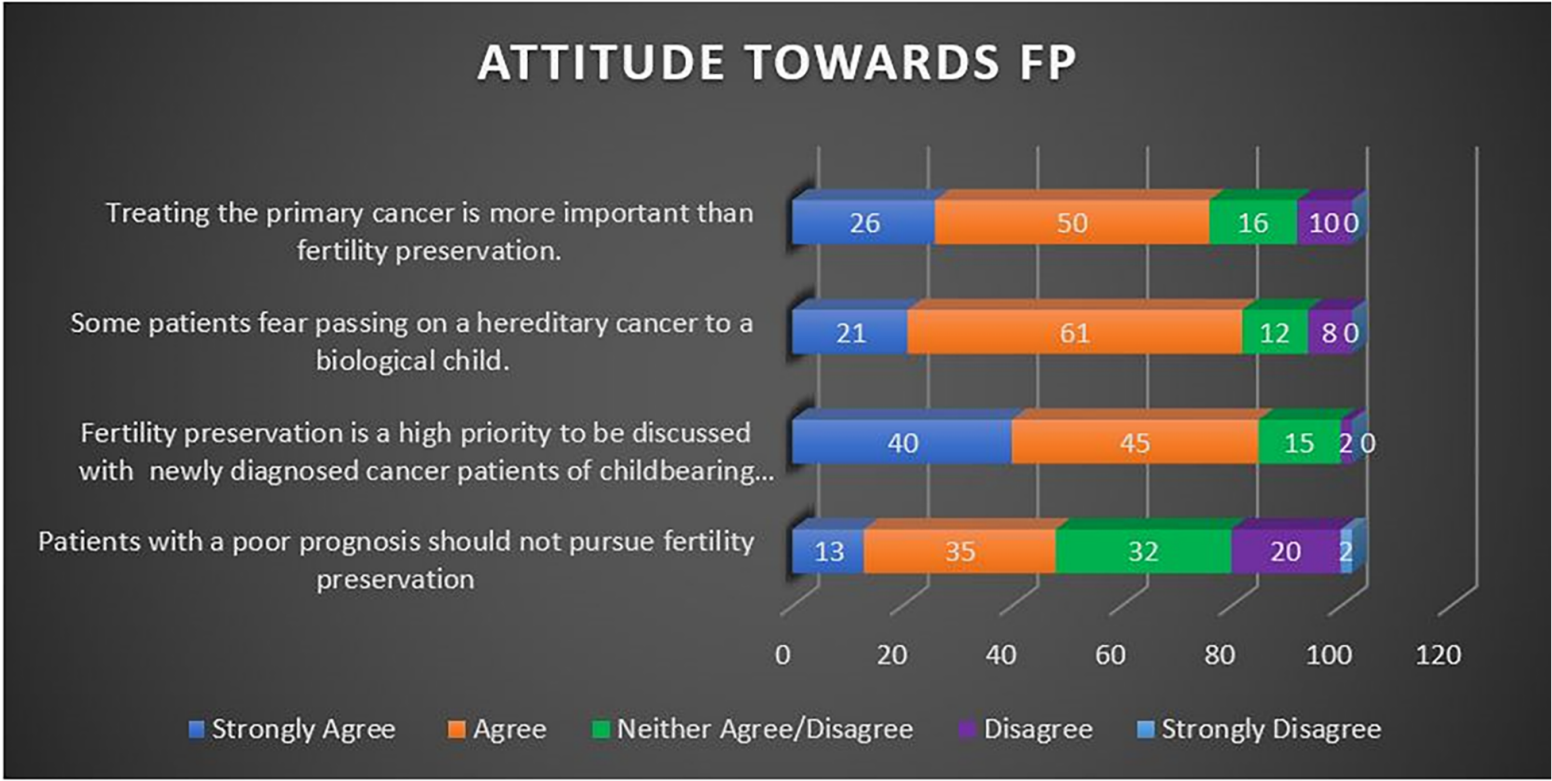
HCPs attittude towards FP.

Most HCPs exhibited favorable attitudes on “FP being a priority to be discussed among newly diagnosed cancer patients” (83.3%) and acknowledged “patient's fear of passing hereditary cancer to biological child” (80.4%). There were conflicting responses when asked to indicate agreement with regards to “patient with poor prognosis pursuing FP”. Most HCPs (54; 52.9%) demonstrated an unfavorable attitude towards FP when dealing with patients with poor prognoses. A staggering 90.2% of HCPs exhibited unfavorable attitudes towards FP in the last item of this domain, whereby they prioritized treatment of primary cancer over FP. HCPs’ attitude towards FP is portrayed in Figure 4. Table 3 shows the overall tabulation of each domain's performance.

### Perception towards fertility preservation (FP) practice

**Figure 5:**
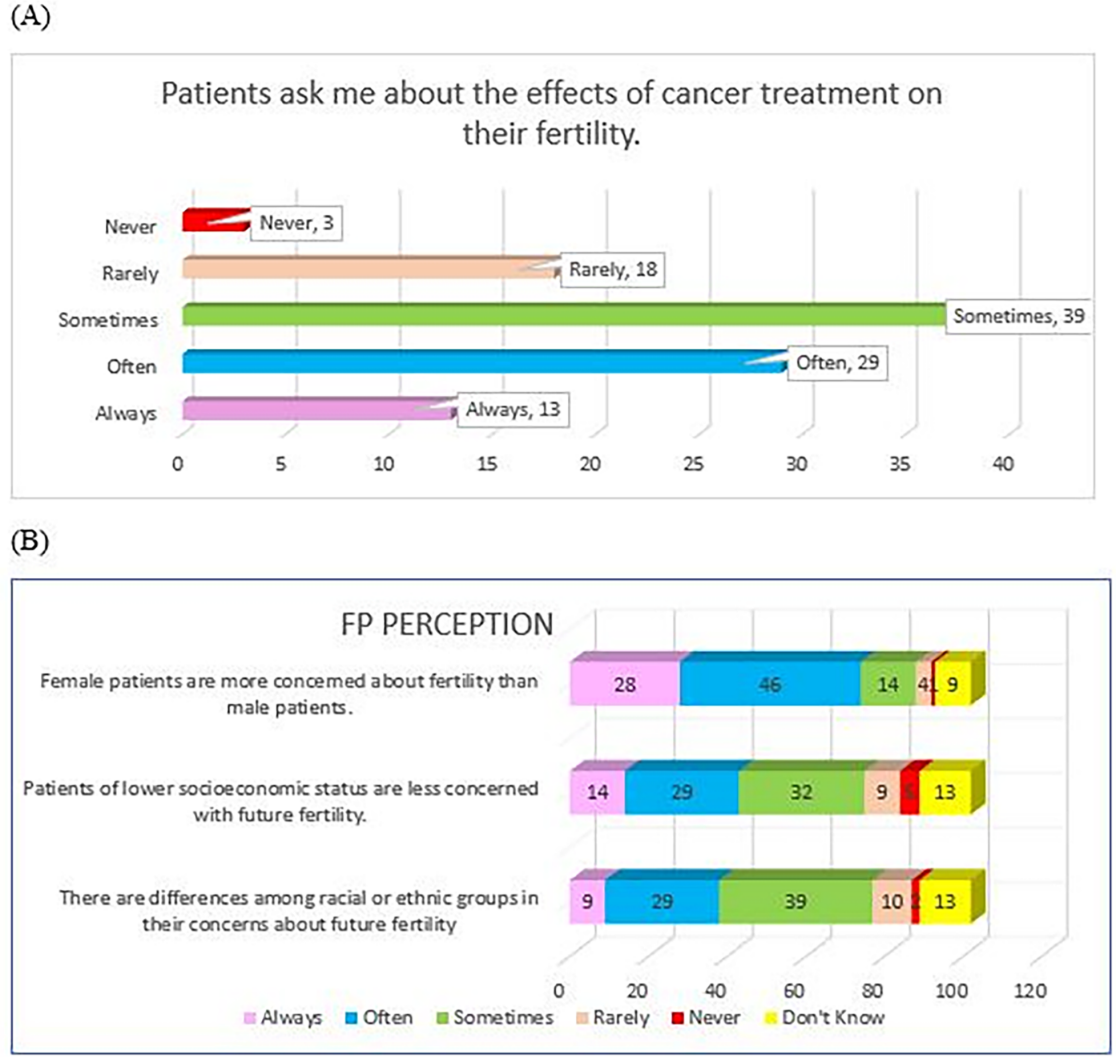
HCP's perception towards FP; **(A)** HCP's perception of patient's concern of fertility effect of cancer treatment, **(B)** HCP's perception pf patient's concern with future fertility according to race/ethnicity, socioeconomics status, and gender.

Overall, majority of HCPs (79.3%) perceived that their patients enquired regarding cancer treatment effects on fertility. Eighty-six HCPs (75.4%) were of the perception that there were differences in future fertility concerns among their patients from different races or ethnicities. Patients of lower socioeconomic status were largely perceived to be less concerned with future fertility (73.5% of HCPs). Collectively, 86.3% of HCPs recognized that their female patients were more concerned about fertility compared to male patients. Responses obtained for this FP Perception domain are detailed in Figures 5A,B. Overall Perception Domain performance is detailed in Table 3.

## Discussion

### Oncofertility service in Malaysia

Malaysia launched its oncofertility services in late 2020. The first oncofertility center in Malaysia is based in a tertiary hospital in Kuala Lumpur. This center, which is nestled in the Assisted Reproductive Center (ARC) caters to the oncofertility needs of adult, children, and adolescent groups for both sexes. The initial part of oncofertility service provision was much dampened by the outbreak of Covid-19 pandemic. It has now gained momentum, and oncofertility services have reached many. Oncofertility services provided by this center include cryopreservation of oocytes, embryos, and sperm, as well as ovarian tissue cryopreservation (OTC). Testicular tissue cryopreservation is not offered in this center, as it is largely experimental for humans. To date, there have been a total of 25 OTC procedures carried out, of which 12 were involving CAYA patients. The current study will focus solely on FP for patients with ovaries (female sex) among the C&A population. This study presents the findings of our survey amongst Malaysian HCPs in a pioneering FP center; assessing their knowledge, attitude, perceived barriers, perception, and overall acceptance towards FP counseling/services for female children and adolescent cohorts.

### Response rate

The response rate that we achieved was 43%, which was comparable to that reported in a previous study by Vadaparampil et al. (14) which was 45% response rate by physicians, and was notably higher as compared to the original study from which we adapted the questionnaire (12) whose response rate was 32%, as well as a few other published physician's survey response rates i.e., 35% as reported by Cunningham et al., and a response rate of 18% in a study by AW. Loren et al. Our slightly higher response rate compared to other reported physicians’ survey response rate could be attributed to the mixed recruitment methods of both; electronic e-mailing as well as physical distribution of hard copies of the questionnaire which improved the overall response.

In terms of non-response rate bias, we examined the characteristics of non-respondents. However as we were unable to collect data for non-responders, the non-bias risk assessment were limited to the only available data which were specialty and gender. It was inferred that the non-responders did not display any typical pattern or significant difference across the different groups of the HCPs sampled which may indicate bias from non-response. Furthermore, the sampled population was homogenous amongst primary HCPs involved in the management of C&A patients, this further reduces the concerns of differential non-response.

### Socio-demographic profile, medical training backgrounds, and clinical practice characteristics

Healthcare providers were majority Malays, females, and Muslims which was seemingly representative of the overall racial, gender, and ethnicity distribution in Malaysia. Although majority of respondents were females which may give an impression of a skewed and non-representative data. In our context of this particular oncofertility center, whereby the specialty involved (O&G, Pediatrics, Oncology and Primary Care) were largely made up of female HCPs across these specialties, thus such findings were evident. A fairly similar trend may be seen in tertiary teaching hospitals too where females are usually more compared to males. This could explain the largely female-populated respondents. HCPs with children and the younger generation of HCPs, (having graduated from medical school in 2011 and later) predominated in this survey compared to prior physician survey studies on FP which involved more senior physicians (12, 13). Based in a tertiary referral center which is also an academic center, it is expected that largely the HCPs would be from the university-affiliated or teaching hospital with multi-specialty practices. Our study involved HCPs across a few specialties managing children and adolescents (gynecology, pediatrics, oncology, and others) in a single center, this is different from previously reported studies assessing physicians’ views on various FP aspects which were conducted to more specialized care centers such as bone marrow transplant centers (15) or amongst a single specialty HCP cohort such as pediatric oncologists *per se* (16), or oncologists (12, 17, 18). Even though this may not be representative of the general HCPs in practice locally, as the FP for children and adolescents service is at the current time, only available in this single center, it is thus considered a fitting respondent cohort. Furthermore, being a referral center, the likelihood of high exposure to a large number of eligible patients for FP was beneficial for study recruitment.

### Female FP knowledge

Seventy-three (71.6%) out of the total HCPs who participated in this survey were knowledgeable concerning female FP services. This figure was slightly lower than the reported accurate physicians’ FP knowledge of 74% (16). It was however higher than that reported by Tress Goodwin et al. which was between 22% and 50%. This could partly be attributed to the efforts of FP knowledge dissemination now in Malaysia via platforms such as webinars as well as an increasing number of talks on FP at various national level congresses, which may have helped in increasing HCPs’ awareness and knowledge of FP. Comparatively, our findings echoed the reported results of studies where treating physicians were generally knowledgeable on FP issues (15). Previous FP studies assessing knowledge were mostly qualitative studies identifying themes, or those comparing knowledge domain components quantitatively, thus hand-in-hand comparison of figures of the overall knowledge status of HCPs may not be feasible in this context. Having said that, a few publications on various aspects of FP discussion among adult oncologists have constantly identified gaps in knowledge as a limiting factor to the conduct of FP discussion (19). A systematic review by Vindrola-Padros et al. evaluating HCP's FP discussion with children, adolescents, and young adults also discovered a lack of knowledge as a leading factor influencing these FP discussions (20). Other authors looking into FP discussion among younger patients also shared similar findings of knowledge gaps among others, pertaining to the available/possible FP options for these young girls. Moreover, lack of knowledge of the FP subject was among themes identified under “personal discomfort” which was found to be associated with physicians’ uptake of FP discussion. Physicians were found to be more willing to take up FP discussions if they personally felt comfortable with the topic (13). Evidence pointed out that being knowledgeable on matters pertaining to FP is a crucial factor in ensuring that FP counseling/discussion is conducted well (8). Knowledge in this context includes knowledge of the various available FP techniques as well as resources and referral places to recommend to patients who are keen to undergo FP procedures (19, 21). On the contrary, a published report by Adams, Hill, and Watson showed conflicting evidence whereby oncologists responding to the survey confessed to having a lack of knowledge and needing more FP information but still expressed being comfortable discussing FP with patients. The author further highlighted that in-depth FP knowledge may not be crucial to enabling a sound discussion on cancer treatment's impact on fertility, but what is most vital is the ability to identify who would be a suitable candidate for FP (18). This poses an interesting view as it contrasts with other reports that emphasized the essence of being knowledgeable to provide thorough FP counseling and discussion with patients; both adults and pediatrics (13, 15, 19–21). It also opens up a horizon to what should ideally be included when HCP counsels a patient regarding FP, furthermore if involving children and adolescents.

### FP practice behaviors

Eventhough, the overall practice behaviors of HCPs with regards to consulting reproductive specialists when faced with fertility issues as well as referrals of patients for FP were good in this study, several crucial aspects in current practices need to be addressed. To start off, this study discovered that a handful of HCPs (between 7% and 15%) were neither regularly consulting nor referring patients for FP albeit, they themselves or the patients having questions or issues with regards to FP; which was a concern. These were similarly reported by previous authors, however at a higher rate; whereby Köhler et al. published that despite pediatric oncologists recognizing that the threat to their fertility was a major concern amongst their patients, only 46% of them referred their male patients, and a lower rate (12%) of pediatric oncologists referred their female patients to fertility specialists, prior to treatment commencement (16). M. Arafa et al. reported fairly similar rates of only 14% referral to fertility specialists amongst oncologists despite them acknowledging and demonstrating positive attitudes towards female FP (17).

### Attitude towards FP

The majority of our HCPs scored poorly in the attitude domain, with 90 (88.2%) found to have an unfavorable attitude towards FP counseling/services. This was high compared to 45% which was the reported attitude of oncologists being unaware of FP options for females (17). G. P. Quinn et al. reported in her study of oncologists’ views on FP discussion with patients that oncologists expressed different attitudes towards FP discussion based on the patient's gender; whereby the attitude was towards not discussing FP among female patients due to limited available FP options for females at that time (21). (This could be since at the time of these previous study publications, oocyte and ovarian tissue cryopreservation were still considered experimental). However, this attitude may have changed over time with more available and established female FP techniques.

It was observed that these unfavorable HCPs’ attitudes were largely classified as such due to two items that were surveyed in the attitude domain. These two items were (i) patients with a poor prognosis should not pursue fertility preservation and (ii) treating the primary cancer is more important than fertility preservation. The lower scores could be attributable to conflicting views or skepticism among HCPs on these issues. These occurrences are relatively common as HCPs are inclined to project their personal values or even beliefs either intentionally or unintentionally during discussions of FP with patients (22, 23). When faced with a patient whose prognosis is poor, the attending HCP would understandably be slapped with the dilemma of discussing or omitting FP discussion, as there are more “pressing” issues at hand, i.e., the aggressive or advanced stage of the patient's disease with poorer prognostication. Approaching the FP issue when dealing with poor prognosis patients in actuality could work in either way; positive or negative; depending on how the FP subject was broached, and also the willingness or acceptance of the patients and/or parents or caregivers. Going down the positive path, discussing FP options, could be seen as a motivating factor for the patients despite a said poorer prognosis and in some patients could be a hint of hope to recover and possibly have offspring in the future, a reason to fight the disease and be strong throughout the ordeal. On the other hand, in a more pessimistic tone, discussing FP with patients of poor prognosis could be seen as insensitive or promising the impossible and for some promoting investing their money in intangible things of the future when it could be directed for medical treatment of current illness and other matters in life. In some practices, poor prognosis patients may be an exclusion criterion for FP to be offered. This is however not true in all circumstances and needs to be tailored on a case-to-case basis. Previously published reports also encountered similar biases in respondents’ feedback to FP discussion amongst poorer prognosis patients. AW. Loren et al. for instance, reported observation of significant differences between transplant oncologists treating pediatrics and adults patients, whereby those managing pediatric cohort were less likely to perceive their patients to be too ill to initiate FP discussion, thus they were more likely to talk about infertility issues even among patients of poor prognosis as compared to adult oncologists (15).

In terms of prioritizing treating primary cancer over FP, professionals’ attitudes that focused on cancer eradication instead of FP were echoed in a few other studies (18, 24). The understanding that opting to pursue FP robs patients of their primary cancer treatment may need to be revisited as there are available FP options that could be employed in patients who could not delay precious therapy commencement timing. Additionally, the time needed to effect FP prior to cancer treatment commencement is short and could be agreed upon with the involvement of a multi-disciplinary team namely an oncologist, reproductive specialist, and others as needed. Prioritizing treating primary cancer over FP if practiced as a blanket rule and not addressed by increasing HCPs’ FP awareness and knowledge, as well as meticulous patient selection may deprive eligible patients of these available FP options.

### Perception of FP

This study found that HCPs’ perception of whose patients were more likely to ask about fertility effects of cancer treatment could be a positive pushing factor for them to effect referral to a reproductive specialist for FP discussion, if not they themselves, initiating the FP discussion. This was similar to findings from a previous study by the author of the original questionnaire (12). AW. Loren also reported in her study that transplant physicians who perceived that their patients were interested in learning more about fertility impact of transplants were more likely to discuss FP (15). Initiating FP discussions with patients who just discovered a cancer diagnosis is challenging, however, failure to initiate these FP discussions before cancer therapy, throughout the continuum of care for fear of adding to the patients’ emotional grievances was associated with significant regret among these cancer survivors upon learning of infertility effects of the treatment (25–27). HCPs in this study perceived that differences in ethnicity or race may affect patient's concerns about future fertility. A study by Al Gaithi et al. reported that 62% of oncologists perceived that gender played a role, whereas 27% of the oncologists perceived females to be more concerned about fertility. The similar study also reported that 67% of oncologists perceived that both lower or higher socioeconomic status affect a patient's attachment to future fertility, while 27% of oncologists perceived this to be true among patients with higher socioeconomic status (28). Apart from the above, perceived uncertainty of the success of FP methods was reported to be a limiting factor to FP practice and discussion (29). This was however not addressed in this study.

### Barriers to FP counselling/services

The main barrier towards FP counseling/service which was voted by our HCPs was patients could not afford FP (30.4%). Cost issues limiting HCPs discussing FP with patients were among the frequently cited FP barriers across a few published studies (12, 13, 19, 20, 30–33). The additional cost in this scenario was not restricted only to funding of the FP procedure but extends also to the ensuing assisted reproductive technologies required as well as tissue storage. Lack of insurance coverage for fertility-related treatments, inclusive of FP in some countries further worsens the patients’ monetary burden (12, 13, 28, 34). Full or partially funded FP procedures by either government agencies or insurance companies would positively influence FP uptake as it would then be more affordable to patients.

The second most common barrier selected by our respondents was limited information on FP to offer patients (17.6%). These findings were consistent across a few previously published studies (19, 20, 35). Takae et al. in their recent publication amongst Asian countries in the year 2019 shared similar FP barrier findings which were “insufficient information” (10). The availability of educational materials on FP to be distributed to patients was thought to be useful in enhancing patient's understanding of FP (28, 36). This in turn would assist decision making which is best suited to each patient's personal values and future aspirations. Sources of FP-related information should also be accessible and conveyed via reliable channels with validated information sharing to enable both HCPs, and patients alike to benefit from these knowledge portals. HCPs should be proactive in ensuring they are equipped with updated information on FP for this C&A cohort, to enable productive sessions of FP discussion. This also emphasizes the dire need for C&A patient-tailored FP information to be made available for use during FP discussion to enhance comprehension and assist FP decision-making. An unmet need exists for a tailored intervention that could provide timely reliable FP information and decision-making assistance for FP counseling in limited clinic consultations (37). Decision aid (DA) was commonly proposed to overcome this unmet need for tailored FP information for C&A to enable effective FP counseling to be conducted within a limited time in the clinic, yet patients would still have the DAs to aid their informed decision-making in terms of FP (37–39). A published study reported the astounding performance of a “fertility-preservation toolkit” in a hospital setting. Its use in FP discussion was demonstrated to markedly enhance the clinician-rated perception of patients’ or their families’ comprehension of fertility information, reported to be as high as 90%. Physicians themselves were 100% satisfied with their conduct of FP discussion using the toolkit (40).

Apart from unaffordable FP cost and lack of FP information to give to patients, among other barriers to FP discussion in this study were similar to those published in previous studies; namely, delay commencing cancer treatment (16, 17), and lack of knowledge on FP referral or resources (12, 21). Our findings of primary FP barriers were different from what was reported by Takae et al. who named “low recognition (of FP for children and adolescents) among medical staff” as the primary barrier to the promotion of FP for children and adolescents which was voted by 9 out of 11 participants (10). It is however interesting to highlight that a particular HCP reported that his/her “treating gynecology malignancy” was a primary barrier to FP counseling/services. Such remarks could be likened to the perception and attitude of prioritizing treating primary cancer over FP consideration, which was reported as a more common finding among adult oncologists who perceived FP as a low priority in their practices compared to the pediatric oncologists who ranked FP as moderate to high amongst their priority (21). This aspect has already been elaborated on above under the discussion for the attitude domain.

### Limitation

Some limitations to our study need to be taken into consideration. (i) Our sampling frame involving a single center may not include all eligible HCPs thus limiting generalization as it may not be representative of overall Malaysian HCPs views. Nonetheless, as this is the only center offering pediatric FP, this sampling frame was deemed appropriate. (ii) Items in the questionnaire involving highly controversial FP issues under the attitude domain tipped the scale towards unfavorable attitude despite good FP knowledge. Nevertheless, it provided an understanding of this variation in attitude and perception, offering valuable indicators for future information requirements and HCP training provisions. (iii) Small number of male HCP respondents and unequal distribution of respondents per specialty renders it inappropriate to explore the probability of gender or specialty differences as the analysis may be lacking sufficient power. Furthermore, the highly female HCPs density at our center (tertiary teaching hospital) across the specialties involved contributed to such findings. Additionally, considering that this was a pilot study in a single center, thus its findings may not be representative of the overall landscape of HCP practices all over Malaysia. Future studies may aim to evaluate these differences in a larger and more homogenous sample. We believe that duplication of this study or a fairly similar approach in a multi-center setting and extension to large oncology centers in the future may provide a better representative of overall HCPs in Malaysia. (iv) This study did not look into HCPs’ sources of knowledge on FP and data on age and job rank were not collected, therefore limiting meaningful comparisons of knowledge, practice behaviors, attitude, and perception at various levels of employment hierarchy. These limitations are however consistent with the limitations of other surveys.

Several implications for future clinical practice can be drawn from the findings of this study. Knowledge dissemination with regards to FP for C&A should be actively promoted at various levels of healthcare systems as an initial step, to consolidate knowledge of this important aspect of the new medicinal frontier. Call for action is critical to address the findings of unfavourable attitude most likely stemming from personal bias with regards to patient selection for FP counseling/service provision. Data and findings from this study will be utilized for the establishment of the first guideline for FP among C&A in Malaysia. It will also intended to steer the development of clinician-based intervention/tools for promoting and assisting FP discussion for C&A patients in hospital settings. Additionally, concerted efforts and mitigation plans could be strategically devised and put forward to combat the C&A FP barriers identified.

### Conclusion

In summary, this survey reflects our HCPs’ current knowledge, practice behaviors, attitudes, and perceptions toward FP counseling/practices for children and adolescents. It also identified the main barriers to FP discussions in clinical practice. Healthcare providers’ knowledge of FP was generally good and similarly was the overall FP practice behavior. Although the overall attitude towards FP was poor, it shed light on biases in clinical judgment affecting negative attitudes towards FP. Mitigating several controversial FP issues via knowledge dissemination and improving awareness of FP, would in time improve HCPs’ attitude, as it appears perception-oriented and not a core belief. The findings of this survey provided valuable insight into barriers towards FP counseling/services which would enable the planning of effective strategies for FP funding assistance as well as emphasizing the dire need to develop age-appropriate pediatric FP-related educational and information materials to assist FP counseling and, subsequently achieve shared decision-making. To conclude, with remedial measures in place, the future of FP services for the pediatric population in Malaysia appears promising.

## Data Availability

The original contributions presented in the study are included in the article, further inquiries can be directed to the corresponding author.
